# How to critically appraise and direct the trajectory of AI development and application in oncology

**DOI:** 10.1016/j.esmorw.2024.100066

**Published:** 2024-08-29

**Authors:** R.S.N. Fehrmann, M. van Kruchten, E.G.E. de Vries

**Affiliations:** Department of Medical Oncology, University Medical Center Groningen, University of Groningen, Groningen, the Netherlands

**Keywords:** artificial intelligence, AI regulatory frameworks

## Abstract

As artificial intelligence (AI) advances, oncologists stand at the forefront of a transformative era in healthcare. AI, which empowers machines to learn from data, make decisions, and carry out tasks typically requiring human intelligence, is revolutionizing our clinical landscape. It promises streamlined workflows, enhanced diagnostic accuracy, and personalized treatments tailored to each patient’s unique profile. In the vast sea of patient data, AI serves as a guiding compass, ensuring no detail is overlooked, amplifying clinical acumen, and refining treatment decisions. However, to ensure AI’s benefits reach patients effectively, it is imperative that oncologists actively guide its development and application. This overview aims to equip oncologists with the tools to critically appraise and influence the trajectory of AI in oncology, ensuring its integration leads to meaningful advances in patient care.

## Highlights

• AI enhances oncology via better diagnostics, personalized treatments, and data.
• AI in healthcare has challenges: privacy, explainability, bias, skills, and regulation.
• EU AI healthcare regulation includes MDR, IVDR, and the AI-Act.
• AI evaluation needs patient-centric trials. Oncologists must define and develop AI.
• AI’s future in oncology needs structured development, evaluation, and education.

**Figure 1:**
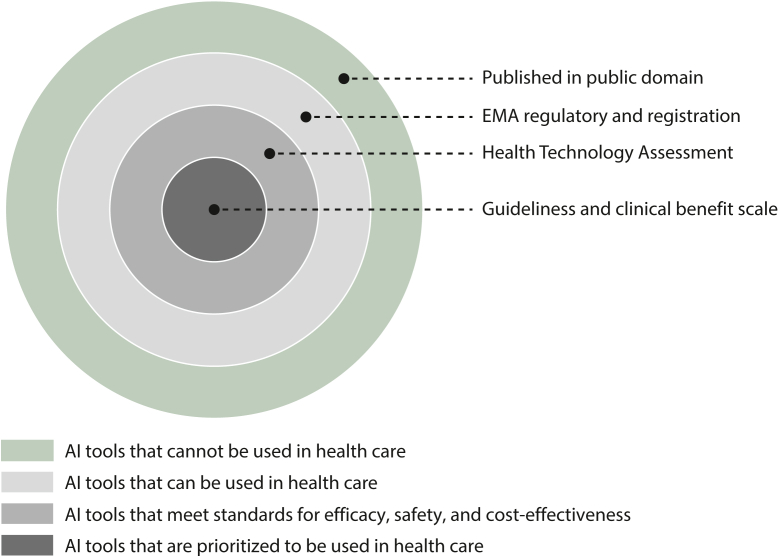
**Prioritizing artificial intelligence (AI) tools for application in oncology.** EMA, European Medicines Agency.

As artificial intelligence (AI) leaps forward, oncologists find themselves at the frontier of a new era in healthcare. AI, which enables machines to learn from data, make decisions, and carry out tasks typically requiring human intelligence, is reshaping our clinical landscape. It promises streamlined workflows,^1^ improved diagnostic accuracy,^2^ and treatments tailored to each patient’s unique profile.^3^ Recently, large language models such as GPT-4 (OpenAI, San Francisco, CA) have gained significant traction. These models are increasingly used for administrative tasks in healthcare. They can summarize clinical information, extract relevant data from physician notes, and enhance the efficiency of healthcare research.^4^^,^^5^ In the vast sea of patient data, AI emerges as our guiding compass, ensuring no detail goes unnoticed, amplifying our clinical acumen, and refining our treatment decisions. However, to ensure AI is providing real benefits to patients, we must steer the wheel. Therefore we offer an overview for oncologists to critically appraise and direct the trajectory of AI development and application in oncology, ensuring its integration translates to meaningful advances in patient care (Figure 1).

In health care, integrating AI brings several challenges to the forefront. First, data privacy and security are critical, as the utilization of sensitive patient data introduces risks of privacy infringement and data breaches.^6^ Second, the lack of explainability in AI, particularly with deep learning models, presents a challenge; their ‘black box’ nature can obscure the decision-making processes of the model, potentially undermining clinicians’ trust and willingness to adopt deep learning models.^7^ Third, there is a risk of bias and inequality; AI models may learn biases present in training data, which can result in unequal treatment or misdiagnoses, disproportionately affecting underrepresented groups in the training data.^8^^,^^9^ Fourth, an overreliance on AI could lead to skill erosion among clinicians, potentially decreasing traditional diagnostic abilities due to the less frequent practice of manual review and interpretation.^10^ Lastly, the integration of AI in clinical settings faces regulatory hurdles, ethical considerations, and the need for clear guidelines to ensure patient safety and equitable care. Considering these challenges, healthcare providers are compelled to seek clarity and guidance through clear legal frameworks, concise guidelines, and tools to navigate the rapidly advancing AI landscape.

Safe access to high-quality, relevant, and unbiased data is key for AI development in healthcare, for which the European Health Data Space (EHDS) will become instrumental. The EHDS is an initiative by the European Union (EU) aimed at defining clear rules for the ethical use of health data for better healthcare delivery, research, innovation, and policymaking. The EHDS will offer safe and secure exchange, use, and reuse of health data while ensuring full compliance with the EU’s high data protection standards (e.g. the General Data Protection Regulation).

In Europe, AI-enabled software or medical devices intended for clinical use must undergo regulatory approval and registration in accordance with the EU Regulation on Medical Devices (MDR) and *In Vitro* Diagnostic Medical Devices (IVDR). However, since the MDR and IVDR were drafted, AI has rapidly advanced. Therefore the European Parliament approved the Artificial Intelligence Act (AI Act) in March 2024 to complement the MDR/IVDR to address the unique challenges and opportunities presented by AI. This AI Act aims to govern the use of AI across various sectors, not just health care, and is designed to ensure that AI used within the EU is safe, transparent, and accountable. The AI Act now clearly defines an AI system as software that is developed with machine learning, logic- and knowledge-based approaches, or statistical methods and can, for a given set of human-defined objectives, generate outputs such as content, predictions, recommendations, or decisions, thereby influencing the environments they interact with. AI systems in health care, even if they do not make direct medical decisions, are classified based on their intended use and potential risk. According to the AI Act, AI systems used in health care are often deemed high risk, as their failure or inaccuracy could pose significant risks to patient safety and treatment outcomes. These high-risk AI systems are subject to stringent regulatory requirements and conformity assessments. They must have an established, documented, and maintained risk management system. They should be built using quality training, validation, and testing datasets. Detailed technical documentation must be prepared before market introduction and regularly updated. For transparency, these systems are required to have logging capabilities during operation. Their design must allow users to understand and correctly use the system’s outputs, and they should facilitate effective human oversight in use. Finally, high-risk AI systems must be accurately, robustly, and securely designed to perform reliably throughout their lifecycle.

Currently, there are 692 United States Food and Drug Administration (FDA)-approved AI-enabled medical devices (updated until 19 October 2023), of which most, 531 (77%), are in the field of radiology.^11^ We identified 146 FDA-approved AI-enabled medical devices applicable in oncology, of which 142 utilize imaging modalities, while only 4 are pathology based, using tumor tissue. The types of applications for these AI-enabled medical devices are summarized in Table 1. In Supplementary Table S1, available at https://doi.org/10.1016/j.esmorw.2024.100066, we provide detailed information for each FDA-registered device, including the FDA submission identifier, device name, company name, application description, modality (type of data used), and a summary of the device’s intended use. The number of AI-enabled medical devices approved by the European Medicines Agency (EMA) is not readily ascertainable. Most FDA approvals for medical devices are issued via the 510(k) clearance pathway.^11^ This procedure, which primarily relies on showing substantial equivalence to existing devices or current manual methods, frequently circumvents the need for clinical trials. Typically, this equivalence is established using retrospective datasets, often sourced from a single institution or clinical trial, which raises questions about the applicability and transferability of such AI devices to real-world patient populations in different institutes. Only 3% of all FDA-approved AI-enabled medical devices are registered based on clinical trials evaluating their performance.^11^ Two reviews provided an overview of prospective randomized controlled trials (RCTs) that evaluated AI-enabled medical devices.^12^^,^^13^ The most recent review (update until 18 August 2023) reported only 84 RCTs.^13^ Most finished RCTs were open label, predominantly evaluated deep learning systems for medical imaging, and were conducted in single-center settings. Most trials were executed in the United States and China, with gastroenterology as the leading focus. Most trials evaluated interventions on diagnostic accuracy-related outcomes without assessing clinically relevant endpoints.

**Table 1:** Summary of the types of applications for artificial intelligence-enabled medical devices applicable in oncology

Application type	*n*
Assisting radiotherapy planning	35
Breast cancer screening/diagnosis	33
Pulmonary nodule detection	21
Characterization of tumors	12
Prostate cancer screening/diagnosis	8
Assisting surgery procedures	7
Polyp detection	7
Characterization of brain tumors	6
Comparing scans	5
Classification of cancer type	3
Assisting biopsies	2
Characterization of liver tumors	2
Thyroid nodule detection	2
Assisting liver ablation procedures	1
Cervical cancer screening/diagnosis	1

Single-center studies within uniform health systems and well-defined populations have inherent limitations in generalizability to broader real-world populations. The ability to generalize AI tools is critical, especially as the data used to train these tools can significantly differ across various populations, healthcare systems (such as primary, secondary, or tertiary care hospitals), or regions. To overcome the concerns regarding the generalizability of AI results, we must strive for international collaborations and multicenter studies to validate AI tools before their widespread implementation. In addition, gathering real-world data and evidence is essential for validating the efficacy and safety of AI applications in diverse clinical settings. It ensures generalizability across populations, supports regulatory compliance, and fosters clinical acceptance. Furthermore, it provides insights into patient outcomes and economic evaluations, while facilitating continuous improvement and adaptability of AI models. The value of real-world data and evidence was exemplified by the evaluation of the Epic Sepsis model: in a large retrospective cohort of ∼30 000 patients, the widely adopted AI sepsis model performed with an area under the curve of 0.63.^14^ Such results challenge the sepsis model’s practical utility in clinical settings.

**Figure 2:**
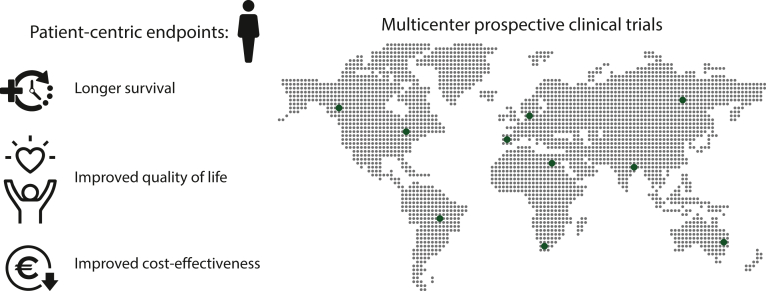
**Evaluating artificial intelligence (AI) tools requires clinical trials focusing on patient-centric outcomes and not just accuracy.** Furthermore, the future of AI in oncology requires an emphasis on multicenter trials and real-world data to ensure the efficacy and reliability of AI tools.

Evaluating AI tools in clinical trials demands the same rigor as in clinical trials with medicines, especially regarding endpoints. Recently, several guidelines and checklists have emerged that are essential for standardizing the reporting and protocol development of AI-based clinical trials, thereby enhancing the credibility and reliability of AI applications in healthcare. These include the Consolidated Standards of Reporting Trials-Artificial Intelligence (CONSORT-AI),^15^ the Standard Protocol Items: Recommendations for Interventional Trials-Artificial Intelligence (SPIRIT-AI),^16^ and the Minimum Information about Clinical Artificial Intelligence Modeling (MI-CLAIM).^17^ Current AI trials predominantly measure accuracy, yet for clinical adoption, we must look beyond. It is essential that AI demonstrates improvements in patient-centric endpoints, such as longer survival, improved quality of life, or improved cost-effectiveness (Figure 2). For instance, a systematic review of AI in detecting colorectal polyps highlighted a critical nuance: despite AI increasing the detection rates, it mainly identified nonadvanced adenomas, leading to unnecessary polypectomies.^18^ This suggests that greater accuracy—often the main endpoint in current AI trials—does not always correlate with clinical benefit and may even have unintended consequences. Thus, we must not wait any longer to incorporate meaningful endpoints in AI trials, ensuring AI’s clinical utility in the future.

In addition to FDA and EMA registration, Health Technology Assessment in Europe serves as an additional layer of evaluation guiding the adoption of AI tools in healthcare. This multidisciplinary process involves an in-depth evaluation, where clinical aspects are scrutinized at the European level to ascertain which AI tools are sufficiently advanced for clinical use, ensuring they adhere to stringent criteria for efficacy, safety, and cost-effectiveness. Concurrently, the assessment of social, economic, organizational, and ethical dimensions is conducted at the national level to ensure alignment with country-specific healthcare frameworks and priorities.

**Figure 3:**
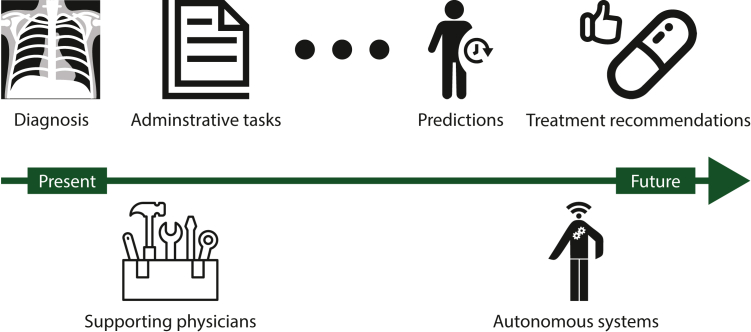
The broad prospects for artificial intelligence models in routine care.

While AI applications in imaging and administrative tasks are gaining traction, there remains a scarcity of tools across the wider spectrum of oncology. As oncologists, we must take a proactive role in defining the AI solutions that will best serve our field and actively participate in their development. AI-enabled medical devices for predictive modeling and treatment recommendations are still more of a future prospect. Furthermore, current AI models are designed to support physicians (not to replace them), but in the future, AI models might operate more autonomously (Figure 3). As outlined above, to ensure the efficacy and reliability of AI tools, they must be scrutinized through prospective, multicenter trials that incorporate real-world data, reflecting the diverse conditions of clinical practice. In addition, a structured evaluation approach akin to guidelines or the ESMO-Magnitude of Clinical Benefit Scale for medicines but now for AI tools could provide an additional robust framework for assessing their clinical value.^19^ Moreover, the rapid advancements in AI necessitate the integration of AI education within the medical curriculum and training for the current practicing physicians. There is a growing need for oncologists who specialize in the utilization of AI in oncology care, ensuring that these emerging technologies are applied effectively in patient management.

In summary, the path forward must include structured development, rigorous evaluation, and comprehensive education to harness AI’s full potential in oncology. By doing so, we can pave the way for AI to enhance patient outcomes and drive the next generation of cancer care.
